# Immediate Effects of Neurodynamic Sliding versus Muscle Stretching on Hamstring Flexibility in Subjects with Short Hamstring Syndrome

**DOI:** 10.1155/2014/127471

**Published:** 2014-04-15

**Authors:** Yolanda Castellote-Caballero, Maríe C. Valenza, Emilio J. Puentedura, César Fernández-de-las-Peñas, Francisco Alburquerque-Sendín

**Affiliations:** ^1^Hospital Virgen de las Nieves, Servicio Andaluz de Salud, Avenida de las Fuerzas Armadas 2, 18014 Granada, Spain; ^2^Department of Physical Therapy, Universidad de Granada, Avenida del Hospicio s/n, 18071 Granada, Spain; ^3^Department of Physical Therapy, School of Allied Health Sciences, University of Nevada Las Vegas, 4505 Maryland Parkway, P.O. Box 453029, Las Vegas, NV 89154-3029, USA; ^4^Department of Physical Therapy, Occupational Therapy, Rehabilitation and Physical Medicine, Universidad Rey Juan Carlos, Avenida de Atenas s/n, Alcorcon, 28922 Madrid, Spain; ^5^Department of Physical Therapy, Universidad de Salamanca, Patio de Escuelas, 137008 Salamanca, Spain

## Abstract

*Background*. Hamstring injuries continue to affect active individuals and although inadequate muscle extensibility remains a commonly accepted factor, little is known about the most effective method to improve flexibility. * Purpose*. To determine if an isolated neurodynamic sciatic sliding technique would improve hamstring flexibility to a greater degree than stretching or a placebo intervention in asymptomatic subjects with short hamstring syndrome (SHS). * Study Design*. Randomized double-blinded controlled trial. *Methods*. One hundred and twenty subjects with SHS were randomized to 1 of 3 groups: neurodynamic sliding, hamstring stretching, and placebo control. Each subject's dominant leg was measured for straight leg raise (SLR) range of motion (ROM) before and after interventions. Data were analyzed with a 3 × 2 mixed model ANOVA followed by simple main effects analyses. * Results*. At the end of the study, more ROM was observed in the Neurodynamic and Stretching groups compared to the Control group and more ROM in the Neurodynamic group compared to Stretching group. * Conclusion*. Findings suggest that a neurodynamic sliding technique will increase hamstring flexibility to a greater degree than static hamstring stretching in healthy subjects with SHS. * Clinical Relevance*. The use of neurodynamic sliding techniques to improve hamstring flexibility in sports may lead to a decreased incidence in injuries; however, this needs to be formally tested.

## 1. Introduction

Injuries to the hamstring musculature are commonplace in many mainstream sports and occupations involving physical activity [1, 2]. They have not declined in recent times and the high rate of recurrence suggests that the current understanding of such injuries remains incomplete [3]. Hamstring muscle strains/tears account for 13–15% of injuries in Australian football [4, 5], 11% of injuries in elite New Zealand cricketers [6], 12–14% of injuries in professional soccer [7, 8], and up to 24% of injuries in Gaelic football [9]. Many predisposing factors for hamstring injury have been suggested within the literature, including insufficient warm-up [10], poor flexibility [11], muscle imbalances [12], neural tension [13], fatigue [14], and previous injuries [15].

Inadequate flexibility within the posterior thigh compartment appears to be one of the more commonly accepted causes of hamstring injuries [2, 16]; however, the evidence for decreased hamstring flexibility as a risk factor remains equivocal [11, 17]. A recent Cochrane review found no evidence for stretching as a sole intervention for prevention of hamstring injury [18], and this has led to the suggestion that decreased flexibility is but one factor in the multifactorial etiology of hamstring strain injury [19].

Despite varying theories for the observed increases in muscle flexibility after the application of stretching, we are lacking in evidence for any credible explanation. In a recent review article, Weppler and Magnusson [20] suggested that such increases in tissue flexibility may result, not from affecting the mechanical properties of the muscle being stretched, but from changes in the individual's perception of stretch or pain. They suggested that the point of limitation in hamstring range may increase, not because of changes within the muscle structure itself but rather because the individual experiencing the stretching may adopt a “new stop point” for limitation in hamstring range based on altered perceptions of stretch and pain. They referred to this as the “sensory theory” and proposed that increases in muscle flexibility after stretching were likely due to the modified sensation [20]. Changes in the mobility of the nervous system (neurodynamics) achieved through movement and stretching could modify such sensations [21–23].

Decreased hamstring flexibility as evidenced by limited range in the passive straight leg raise test (SLR) could be due to altered neurodynamics affecting the sciatic, tibial, and common fibular nerves [24]. Altered posterior lower extremity neurodynamics could arguably influence resting muscle length and lead to changes in the perception of stretch or pain [25]. Providing movement or stretching could lead to changes in the neurodynamics and modification of sensation and could help to explain the observed increase in flexibility.

The mechanosensitivity of the neural structures in the posterior leg, thigh, buttock, and vertebral canal may play a part in determining the flexibility of the hamstring muscles. Protective muscle contraction of the hamstring muscles found in the presence of neural mechanosensitivity [26, 27] may account for hamstring tightness and thereby predispose the muscle to subsequent strain injury. Neurodynamic sliding interventions are thought to decrease neural mechanosensitivity [26, 28, 29] and it is possible that the inclusion of these interventions in the management of hamstring flexibility could be beneficial.

In a recent pilot study involving 28 male soccer players, our research team was able to demonstrate that a neurodynamic sliding intervention led to a short-term increase in hamstring flexibility [30]. Findings from that study were limited by a small sample size, inclusion of young males only, and also because the experimental group was compared to a control group that received no intervention. Despite these shortcomings, the study did suggest that neurodynamic treatment can significantly increase hamstring flexibility in a young male athletic population and proposed that future research compares neurodynamic techniques with other interventions in a broader population of subjects.

Therefore, the aim of this study was to examine the immediate effects of a neurodynamic sciatic sliding technique, hamstring stretching, and placebo control intervention in asymptomatic subjects with decreased hamstring flexibility or short hamstring syndrome. We hypothesized that an isolated neurodynamic sciatic sliding technique would improve range of motion, assessed by passive straight leg raise test (SLR), greater than hamstring stretching or placebo in the short term. Findings from this study may provide further the evidence for the relevance of neural tissues in determining range of motion and may indicate benefits for adding neural mobilization techniques to the rehabilitation and/or prevention of hamstring injuries.

## 2. Methods

### 2.1. Subjects

**Figure 1:**
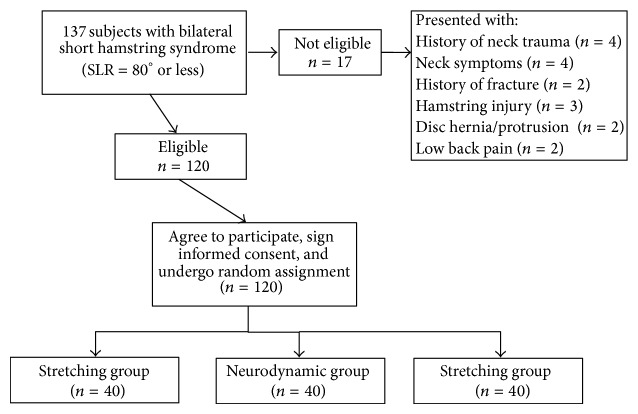
CONSORT flow diagram of patient recruitment and retention.

We recruited a sample of 120 subjects (60 female; mean age 33.4 ± 7.4, range 20–45) who exhibited bilateral short hamstring syndrome (SLR test = 80° or less) [31–33]. Sample size was calculated using Ene 3.0 software (Autonomic University of Barcelona, Spain) and calculations were based on detecting between-group mean differences of 7° at postdata [30]. Assuming a standard deviation of 8° when comparing 2 means, an alpha level of 0.05, and desired power of 90%, a sample size of 29 subjects per group was generated. We increased the sample size by 25% (40 per group) to increase statistical power. Exclusion criteria were hamstring injury within the past year, exceeding 80° in the initial SLR test, verbal report of performing regular lower extremity muscle stretching exercises, history of neck trauma (whiplash), neck symptoms, history of fracture in any part of the body, history of growth disorders, history of neurological or orthopedic disorders, diagnosis of herniated disk, low back pain in the last 6 months, and body mass index (BMI) lower than 20 Kg/cm^2^ or higher than 30 Kg/cm^2^. We chose the BMI range as an exclusion criterion to allow for better identification of body landmarks and introduce some degree of homogeneity for subject body type. Subjects were recruited from the general population via advertisements in local newspapers. All subjects signed an informed consent before they were included in the study, and all procedures were conducted according to the Declaration of Helsinki. The period of recruitment was from January 2009 to June 2011.  Figure 1 provides a flowchart of subject recruitment during the study.

### 2.2. Measurement of Hamstring Flexibility

**Figure 2:**
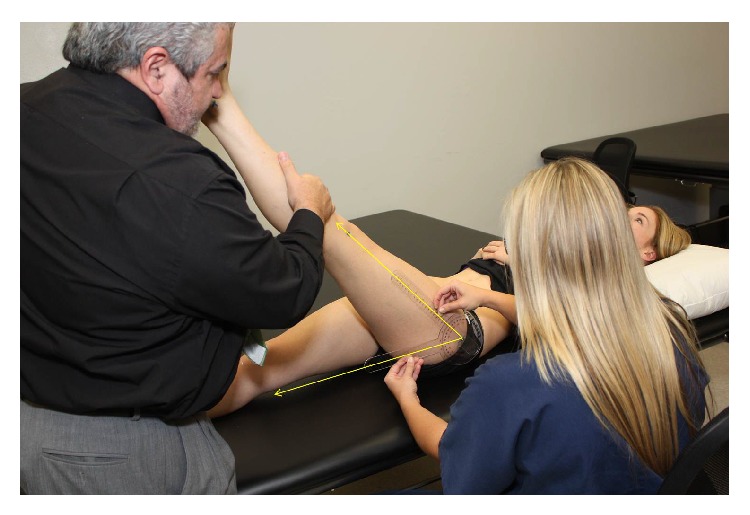
Measurement of range during passive straight leg raise test was performed by trained examiners who were blinded to subject group assignment.

All physical measurements were obtained by a pair of trained examiners who were blinded to each subject's group allocation. The passive SLR test was used to determine changes in hamstring muscle flexibility and has demonstrated high interobserver reliability (0.94–0.96) [34, 35]. Each test was performed with the subject supine wearing shorts or underwear, and the following bony landmarks were identified and labeled with a marker: the anterior superior iliac spine (ASIS), greater trochanter and lateral epicondyle of the femur, and the head of the fibula and the fibular malleolus. The passive SLR test was recorded 3 times for each subject using a universal goniometer. One examiner performed the passive SLR by keeping the knee in full extension and the ankle in neutral plantarflexion-dorsiflexion. Full ankle dorsiflexion was avoided to prevent calf muscle stiffness or pain (gastrocnemius and soleus) from confounding the sensation of hamstring stiffness and pain which would signal the limit of the SLR test. The examiner would hold the talus and avoid any hip rotation during flexion of the hip as they lifted the subject's lower limb until he or she first complained of stiffness or pain in the region of the thigh, bent his/her knee, or began to swing into a posterior pelvic tilt (noted as movement of the ASIS). The second examiner placed the axis of the goniometer over the mark on the greater trochanter of the femur. The stationary arm of the goniometer was placed parallel to the table and checked with a level, and the moving arm was placed in the line between the head of the fibula and the fibular malleolus, and the degree of elevation of the straight leg was then noted (Figure 2). Within-session intra-rater reliability was established on the first 10 subjects as sufficient for clinical measurement (ICC = 0.96); and although less relevant to calculation of responsiveness data and subsequent interpretation of the study findings, between-session intra-rater reliability was similarly established as sufficient (ICC = 0.94). The average of 3 measurements was obtained for each subject and used to determine changes in hamstring muscle flexibility.

### 2.3. Randomization

Subjects were randomly divided into 3 blocks of 40, using a simple random distribution (http://www.randomization.com/) into the 3 intervention groups: Stretching, Neurodynamic, and Control. The Stretching group would receive static passive stretches to their hamstring muscles; the Neurodynamic group would receive neurodynamic sliders; and the Control group would receive passive mobilization of their intrinsic foot joints as placebo. These interventions were performed on each subject's dominant leg; the duration of each intervention was standardized, and all interventions were provided by a therapist who remained blinded to the SLR measurements. Subjects were informed that the intervention being provided to them was a relaxation technique that was thought to improve SLR comfort and range.

### 2.4. Interventions

#### 2.4.1. Passive Stretching Technique

**Figure 3:**
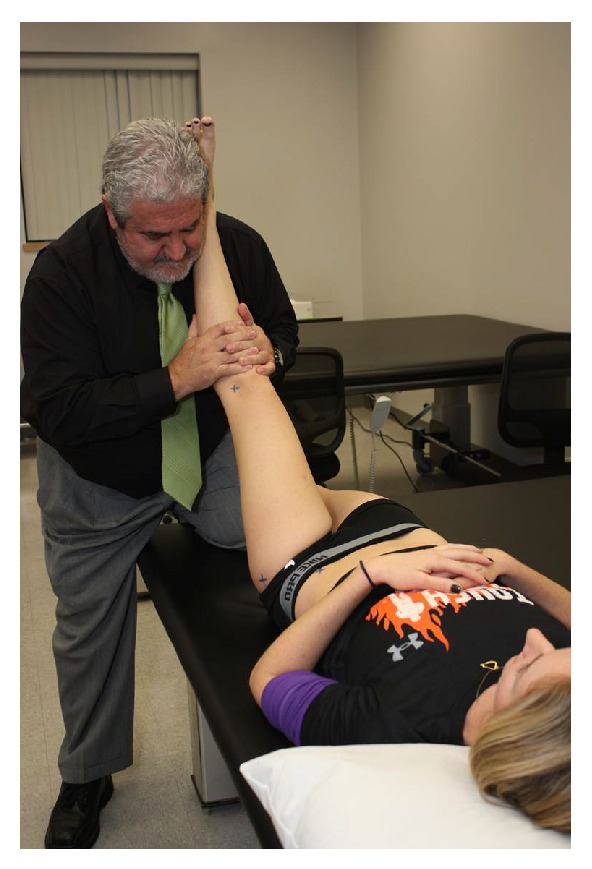
Static stretching of the hamstring muscles was performed for 30 seconds, 6 times on their dominant leg for a total stretching time of 180 seconds.

Subjects in the Stretching group received passive stretching of the hamstring muscles in their dominant leg. While lying supine, a researcher who was blinded to SLR test measures would passively position the subject into the SLR position (hip in flexion, knee in extension, and ankle in neutral) without pain/discomfort to the point where resistance to movement was first noted (Figure 3). This position was then maintained for 30 seconds [36, 37] and repeated further 5 times. During the 30 second stretches, the therapist monitored the subjects to ensure they did not make any compensation that could modify the stretching position. Each subject had a total of 180 seconds of stretching on their lower extremity.

#### 2.4.2. Neurodynamic Sliding Technique

**Figure 4:**
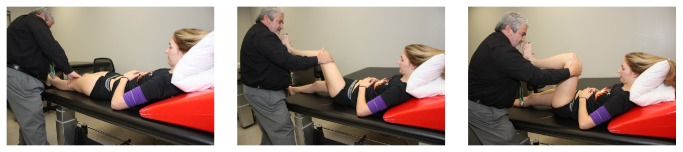
Neurodynamic sciatic slider technique was performed by alternating hip flexion, knee flexion, and ankle dorsiflexion with hip extension, knee extension, and ankle plantarflexion while the subject's cervical and thoracic spine were maintained in flexion. Movements were performed for 180 seconds on their dominant leg.

Subjects in the Neurodynamic group received sciatic neurodynamic sliders, performed in supine. The objective of the technique is to produce a sliding movement of neural (sciatic) structures relative to their adjacent tissues [21, 38]. Sliders involve the application of movement/stress to the nervous system proximally while releasing movement/stress distally and then reversing the sequence. Recent research has shown that sliders actually result in greater excursion than simply stretching the nerve [39]. Subjects were supine with their neck and thoracic spine supported in a forward flexed position. Concurrent hip and knee flexion were alternated dynamically with concurrent hip and knee extension (Figure 4). The therapist alternated the combination of movement depending on the tissue resistance level. This combination of movements was performed for 180 seconds on their dominant lower extremity [40].

#### 2.4.3. Placebo Technique

**Figure 5:**
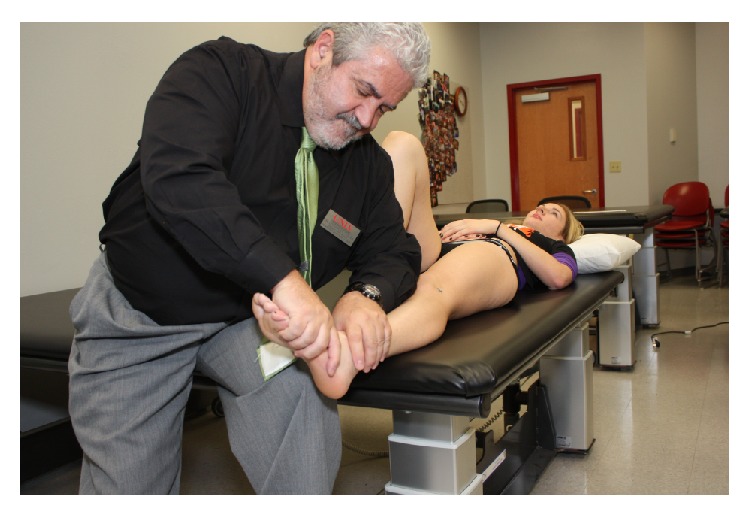
Passive mobilization of the intrinsic foot joints with the subject in supine lying. Passive movements were applied for 180 seconds to the dominant foot.

Subjects in the Control group received passive mobilization of the intrinsic foot joints while being in the supine position. This was chosen as placebo intervention due to the absence of anatomic and/or physiological relations between this region and technique and the hamstring muscles and their stretching positions. Passive movements applied in a randomized order were supination, pronation, abduction, adduction, flexion, and extension (Figure 5). Subjects were given 180 seconds of mobilization to their dominant foot.

As well as standardizing the total treatment times for subjects in each group, preservation of blinding for the study was maintained by standardizing the interval between the SLR assessments for all subjects. No subject reported any persistent discomfort or pain associated with their participation in the study.

### 2.5. Statistical Analysis

Descriptive statistics (mean, standard deviation, 95% CI) were calculated for pretest and posttest SLR averaged values for the 3 groups. SLR responsiveness data were calculated using the between-session ICC established in this study (ICC = 0.96) and the formulae SEM = SD × √(1 − ICC) [41] and MDC_95_ = 1.96 × √2 × SEM [42]. To analyze the difference in pre-/postintervention between groups on hamstring extensibility (SLR), a 3 × 2 mixed model ANOVA was performed, with groups (Stretching, Neurodynamic and Control) as the between-subjects variable, and time (time: pre and post) as the within-subjects variable. The hypothesis of interest was the group × time interaction. Simple main effects analyses with a Bonferroni corrected alpha would be utilized if an interaction was observed. Within-group effect size was calculated using Cohen *d* coefficient (*d*) [43]. An effect size greater than 0.8 was considered large, around 0.5 was moderate, and less than 0.2 was small. All statistical analyses were performed using SPSS version 21.

## 3. Results

There were no significant differences in baseline characteristics between groups at the beginning of the study (Table 1). Descriptive statistics are provided in Table 2. Standard deviations for SLR measurements ranged from 3.7 to 8.0°. This allowed us to calculate a range of SEM from 1.1 to 2.4° and subsequently a range for the MDC_95_ of 3.1–6.6°. The Neurodynamic group had a pretest mean range of 59.8° (95% CI: 58.1–61.3°) and posttest mean range of 69.7° (95% CI: 68.5–70.9°). The Stretching group had a pretest mean range of 59.9° (95% CI: 57.7–62.2°) and posttest mean range of 65.5° (95% CI: 62.9–68.0°). Finally, the Control group had a pretest mean range of 59.4° (95% CI: 57.5–61.2°) and posttest mean range of 59.4° (95% CI: 57.6–61.1°). The before and after differences in mean SLR values exceeded the upper limit of this MDC_95_ range for the Neurodynamic group (9.9°) but not for the Stretching group (5.5°) or the Control group (0.03°) (Table 2).

**Figure 6:**
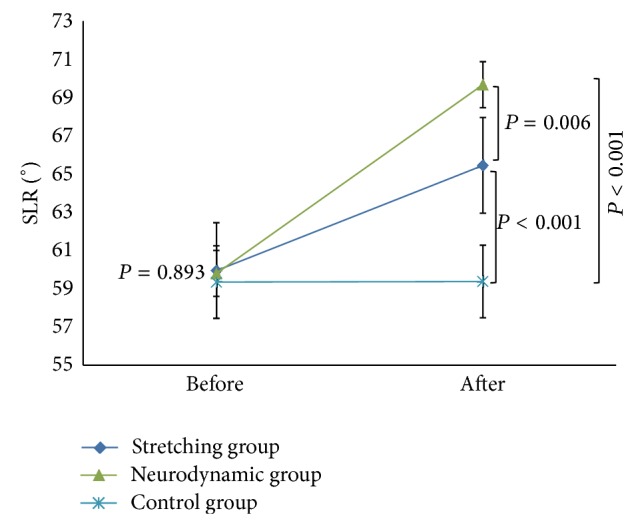
Before and after mean straight leg raise (SLR) values (°) with 95% confidence intervals of hamstring extensibility among the three groups.

A statistically significant interaction was observed, *F*(2,117) = 313.715, *P* < 0.001 (*η*_*p*_^2^ = 0.843) (Figure 6). There was no difference between the 3 groups at the start, *P* = 0.893; however, at the end of the study, the groups were significantly different. Mean SLR values were significantly higher for both the Neurodynamic and Stretching groups compared to the Control group (*P* < 0.001) and for the Neurodynamic group compared to the Stretching group (*P* = 0.006). Both the Neurodynamic and Stretching groups significantly improved ROM compared to their baseline values (*P* < 0.001), whereas there was no significant improvement noted for the Control group (*P* = 0.800). The effect sizes were large for Neurodynamic group (*d* = 2.36), moderate for Stretching group (*d* = 0.73), and very small for Control group (*d* < 0.01).

## 4. Discussion

Results from this study show that an isolated neurodynamic intervention provides a greater immediate increase in passive SLR range of motion than static hamstring stretching in subjects with short hamstring syndrome. Although both interventions were more effective in increasing SLR range than the placebo control, only the neurodynamic intervention group demonstrated before and after differences in mean SLR which exceeded the MDC_95_ upper limit of 6.6°. The results confirmed our initial hypothesis that an isolated neurodynamic sciatic sliding technique would provide a greater immediate improvement in hip flexion, assessed by passive SLR, than hamstring stretching or placebo.

Increasing hamstring flexibility has been suggested as an important factor in the treatment and prevention of lower extremity overuse injuries [44, 45]. Much of the research on increasing hamstring flexibility has focused on the varying modes of stretching, such as proprioceptive neuromuscular facilitation (PNF) [46, 47], static stretching [46–48], plyometric stretching, and ballistic stretching [49]. They have also compared differing stretch intensities [25] and frequencies [50]. Very few studies have examined the effect of neurodynamic interventions on hamstring flexibility [24, 30, 40] and the results of this study can be seen as adding further evidence for the potential role of neural tissue mechanosensitivity in limiting the SLR.

A new conceptual model showing an interrelationship between the different factors involved in hamstring strains may provide a better understanding of this multifactorial injury and therefore improve its prevention and prediction methods [51]. Neural tissue mechanosensitivity presenting clinically as tightness in the hamstrings is a plausible yet only retrospectively studied potential risk factor for and/or potential differential diagnosis to be considered in, hamstring strain injury [52]. The “sensory theory” proposed by Weppler and Magnusson suggests that muscle flexibility and its response to sudden stretch have more to do with perceptions of stretch and pain (sensation) than the biomechanical effects on muscle tissue itself [20].

This proposal was supported in a study by Aparicio and others which demonstrated that a suboccipital muscle inhibition technique altered hamstring muscle flexibility when compared to a placebo intervention [53]. The authors measured hamstring flexibility in 3 ways (forward flexion distance test, straight leg raise test, and popliteal angle test) and found significant before and after differences on all 3 measures for the suboccipital muscle inhibition technique but not for the placebo intervention. The fact that such a distant technique (suboccipital region) could have an immediate effect on the flexibility in the hamstrings may lend support to the “sensory theory” limiting flexibility of the posterior thigh structures. It seems reasonable to attribute the observed increase in hamstring tissue flexibility following the suboccipital muscle inhibition technique to changes in the subjects' perceptions of stretch or pain associated with the 3 flexibility measures.

The results of this study demonstrate a mean increase in the hip flexion range of the SLR for the neurodynamic group of 9.86° which compares favorably with other studies. Castellote-Caballero and others reported mean increases in SLR of 9.4° following a similar neurodynamic slider technique [30], and Aparicio et al. reported mean increases of 5.9° for the right SLR and 5.5° for the left SLR following application of the suboccipital muscle inhibition technique [53]. Mendez-Sanchez and others completed a pilot study on young healthy male soccer players and found mean increases of only 3.7° in the right SLR and 2.2° in the left SLR after sustained hamstring stretching intervention [40]. When they added a neurodynamic slider technique to the sustained hamstring stretching intervention, they found greater mean increases of 6.2° in the right SLR and 6.3° in the left SLR [40]. Finally, Hopper et al. reported mean increases of only 4.7° in SLR after the application of massage techniques to the hamstring musculature [54].

It has been suggested that stretching of the hamstring musculature to improve tissue flexibility may reduce the number of leg overuse injuries after exercise [44], although further high quality studies are needed [55]. While some theories explaining the therapeutic effects of muscle stretching suggest there is alteration of the viscoelastic properties of muscles, studies have shown the importance of distinguishing between real and apparent increases in muscle flexibility [37, 56]. Observed changes in SLR following interventions may be more associated with increased tolerance to the uncomfortable stretch sensation rather than true changes to muscle elasticity [56]. Although the results from this study do not provide information on the mechanisms for the observed changes, they do suggest that neurodynamic interventions can significantly increase SLR more than static stretching in the short term in healthy subjects with short hamstring syndrome.

### 4.1. Limitations

This study only examined immediate effects of a single episode and the lack of longer term follow-up should be considered. It is not known how long the observed increase in hamstring flexibility might have lasted. Furthermore, it is not known if repetition and an appropriate dosage of the neurodynamic interventions over time might lead to longer lasting effects. Finally, we did not conduct any long-term follow-up to determine if the observed changes in flexibility might have resulted in any change in incidence of hamstring injuries in these subjects with short hamstring syndrome.

## 5. Conclusion

Findings from this study indicate that a neurodynamic sliding intervention will increase short-term hamstring flexibility as measured by the passive SLR to a greater degree than static hamstring stretching in healthy subjects with short hamstring syndrome. Future research should look at longer term results and assess the effect of combining neurodynamic techniques with other interventions.

## Figures and Tables

**Table 1:** Baseline sample characteristics.

	Stretching group	Neurodynamic group	Control group	*P* values
	*n* = 40	*n* = 40	*n* = 40	
Gender (female)	20 (50%)	20 (50%)	20 (50%)	1.00^a^
Age (years)	33.9 ± 7.44	33.7 ± 7.68	32.7 ± 7.08	0.75^b^
Weight (kg)	69.8 ± 12.93	68.9 ± 11.09	68.4 ± 10.98	0.87^b^
Height (cm)	170.9 ± 7.75	171.4 ± 7.17	170.7 ± 6.46	0.88^b^
BMI (kg/cm^2^)	23.7 ± 2.63	23.3 ± 2.10	23.3 ± 2.28	0.72^b^

Values are expressed as mean ± standard deviation.

^a^Chi-square.

^b^ANOVA.

BMI: Body mass index.

**Table 2:** Mean passive straight leg raise test (SLR) values pre- and postintervention for each of the 3 groups with associated standard deviations, mean differences over time, and associated 95% confidence intervals (CI).

Intervention	Time	Mean ± SD	Difference between before and after ± SD	95 % CI of the difference	
				Lower bound	Upper bound
Neurodynamic group	Pre	59.8 ± 4.70	9.86 ± 2.51∗	9.07	10.68
	Post	69.7 ± 3.69			

Stretching group	Pre	59.9 ± 6.99	5.50 ± 1.62∗	4.98	6.02
	Post	65.5 ± 7.97			

Control group	Pre	59.4 ± 5.68	0.03 ± 0.62	−0.17	0.22
	Post	59.4 ± 5.45			

All measurements are in degrees.

^*^ *P* < 0.001.
